# Early Marine Migration Patterns of Wild Coastal Cutthroat Trout (*Oncorhynchus clarki clarki*), Steelhead Trout (*Oncorhynchus mykiss*), and Their Hybrids

**DOI:** 10.1371/journal.pone.0012881

**Published:** 2010-09-20

**Authors:** Megan E. Moore, Fred A. Goetz, Donald M. Van Doornik, Eugene P. Tezak, Thomas P. Quinn, Jose J. Reyes-Tomassini, Barry A. Berejikian

**Affiliations:** 1 Resource Enhancement and Utilization Technologies Division, Northwest Fisheries Science Center, National Oceanic and Atmospheric Administration Fisheries, Manchester Research Station, Manchester, Washington, United States of America; 2 School of Aquatic and Fishery Sciences, University of Washington, Seattle, Washington, United States of America; 3 Conservation Biology Division, Northwest Fisheries Science Center, National Oceanic and Atmospheric Administration Fisheries, Manchester Research Station, Manchester, Washington, United States of America; University of Plymouth, United Kingdom

## Abstract

**Background:**

Hybridization between coastal cutthroat trout (*Oncorhynchus clarki clarki*) and steelhead or rainbow trout (*Oncorhynchus mykiss*) has been documented in several streams along the North American west coast. The two species occupy similar freshwater habitats but the anadromous forms differ greatly in the duration of marine residence and migration patterns at sea. Intermediate morphological, physiological, and performance traits have been reported for hybrids but little information has been published comparing the behavior of hybrids to the pure species.

**Methodology/Principal Findings:**

This study used acoustic telemetry to record the movements of 52 cutthroat, 42 steelhead x cutthroat hybrids, and 89 steelhead smolts, all wild, that migrated from Big Beef Creek into Hood Canal (Puget Sound, Washington). Various spatial and temporal metrics were used to compare the behavior of the pure species to their hybrids. Median hybrid residence time, estuary time, and tortuosity values were intermediate compared to the pure species. The median total track distance was greater for hybrids than for either cutthroat or steelhead. At the end of each track, most steelhead (80%) were located near or north of the Hood Canal, as expected for this seaward migrating species, whereas most cutthroat (89%) were within 8 kilometers of the estuary. Most hybrids (70%) were detected leaving Hood Canal, though a substantial percentage (20%) remained near the Big Beef Creek estuary. More hybrids (7.5%) than pure cutthroat (4.5%) or steelhead (0.0%) were last detected in the southern reaches of Hood Canal.

**Conclusions/Significance:**

Given the similarity in freshwater ecology between the species, differences in marine ecology may play an important role in maintaining species integrity in areas of sympatry.

## Introduction

Animal hybridization may represent either a serious conservation concern [1]–[3] or an opportunity for increased population genetic diversity [4], [5]. The hazard or value of hybridization between animal species may depend on the extent of introgression, which is the mixing of gene pools through fertile hybrid progeny. Introgressive hybridization, brought about by removal or breakdown of isolating mechanisms or by species introductions, may result in hybrid swarms and subsequent extinction of native genotypes [2], [6]. In other cases, however, hybridization may increase evolutionary potential through elevated genetic variability and the introduction of novel genetic combinations [7]. Enhanced levels of variability in turn could allow organisms to better respond to environmental change, and to thus evolve more rapidly than through mutation alone [5]. Hybridization has long been recognized as an important evolutionary mechanism for the origin of new species of plants but has only recently been recognized as an important evolutionary mechanism in animals [4], [5], [7].

The ecological circumstances and context of each hybridization case likely determine whether hybridizing species are at risk of extinction or actively involved in the process of speciation. Anthropogenic environmental change has been linked to several cases of vertebrate hybridization and subsequent species collapse [6]. In these situations, human impacts on animal habitats facilitate breakdowns in spatial, temporal, and behavioral isolating mechanisms of previously distinct species [8], [9]. Loss of native species diversity and local adaptation have also occurred through hybridization between native and introduced species [1]. Among fishes, introduced rainbow trout (*Oncorhynchus mykiss*) commonly hybridize with populations of cutthroat trout (*O. clarki*) that did not evolve in sympatry [10]. In other examples, though, hybrid taxa can evolve to create a species genetically distinct from parental taxa [11], [12]. Indeed, all hybrid progeny are not inferior. Rather, hybrids are occasionally more fit than pure parental individuals [13], can be capable of exploiting habitat unavailable to parents [14], and can even displace one of the parental species in an original habitat [15]. Determining the causes and consequences of animal hybridization is not only pivotal to understanding speciation and the definition of a species, but more immediately to effective management of interbreeding species and their habitats.

Hybridization of vertebrates is quite common in nature, especially among freshwater fishes [16]. Hybridization between coastal cutthroat trout (hereafter ‘cutthroat’) and anadromous (migrating from saltwater to spawn in freshwater) rainbow trout (hereafter ‘steelhead’) is well-documented and widespread in streams along the west coast of North America [17]–[24], yet causes and consequences of interbreeding between these species are not well understood. Coastal cutthroat geographic distribution extends from Humboldt Bay, CA to Prince William Sound, AK [25], which largely overlaps with steelhead distribution (Mexico/US border to the Alaskan Peninsula [26]). The two species are thought to have diverged from a common ancestor only 2 million years ago [27]. Most biologists assume that spatial and temporal isolation of spawning behavior have maintained genetic integrity [25], [27], though overlaps in both spawning habitat and timing commonly occur [17], [20]. In contrast to inland subspecies, hybridizing coastal populations of cutthroat and steelhead exhibit low levels of introgression and do not form hybrid swarms [17], [18], [22], but see [28]. Patterns of hybridization appear to be rather consistent, as opposed to episodic [20], and molecular genetic evidence of backcrossed individuals at many study sites provides proof of hybrid viability and of some level of reproductive success [18], [22], [29]. However, most studies of hybridization between anadromous forms of cutthroat and steelhead support the hypothesis that environmental and/or behavioral factors limit complete introgression through a reduction in hybrid fitness [17], [22], [29].

The general life histories of anadromous cutthroat and steelhead in freshwater are similar. Adults of both species spawn in freshwater streams, primarily during the spring months (March–June). Juvenile fish typically remain in freshwater for two to three years before smolting and emigrating to saltwater [30], [31]. Coastal populations of both species usually include individuals that do not emigrate but remain in freshwater for their entire life cycle. This “resident” life history is more common in cutthroat than in steelhead. The two species broadly overlap in stream habitat use patterns, though juvenile steelhead occupy waters with higher velocities than do cutthroat [32]. In contrast to the similarities in freshwater ecology, the marine migration patterns differ substantially between the species. Steelhead spend little time in estuaries, migrate long distances from their natal streams, and spend 1–3 years in the ocean before returning to freshwater [26]. Cutthroat, on the other hand, remain closer to shore upon ocean entry, migrate much shorter distances, and generally spend only summer and fall months in the ocean before returning to overwinter in freshwater [25].

Documentation of natural hybridization between cutthroat and steelhead has been based primarily on the use of molecular genetic markers and examination of pre-smolt juvenile specimens collected in freshwater. Only a few studies have examined hybrid performance or behavior. Hybrid swimming speed and morphology [33] and hybrid aggression [18] have been compared to the same characteristics of pure species, generally finding hybrid levels of each trait to be intermediate to those of pure species. Attempts to assess the fitness of cutthroat x steelhead hybrid parr by comparing proportions of hybrids in relation to pure species at age-0 and age-1, showed no clear pattern [28]. A number of studies have suggested that hybrid individuals are selected against during the marine life history stage, largely due to the lack of adult hybrids observed in nature [17], [18], [20].

In this paper, we report on the early marine migration patterns of naturally-produced hybrids with comparable information on wild steelhead and cutthroat from the same river. We initially designed parallel studies of steelhead [34] and cutthroat [35] but took fin clips for DNA analysis to validate the visual identification of the species. The incidence of hybrids was large enough to enable comparisons of selected aspects of migratory behavior.

## Methods

This study was permitted by the National Oceanic and Atmospheric Administration (NOAA), and all Endangered Species Act consultation requirements were met. Appropriate scientific collection permits were obtained from the Washington Department of Fish and Wildlife.

### Smolt Collection and Tagging

Sixty-six putative coastal cutthroat trout smolts and 117 putative steelhead trout smolts were collected during their downstream migration in spring (mid-April to late May) of 2006, 2007, and 2008 at a weir immediately upstream of the Big Beef Creek estuary (river kilometer (rkm) 0.05) near Seabeck, Washington. Fish were visually identified based on published descriptors of Pacific Northwest steelhead and cutthroat [36]. All specimens were identified in the field as either a steelhead (maxillary does not extend past the eye, no hyoid mark under the lower jaw) or a cutthroat (maxillary extends past the eye, presence of hyoid mark under the lower jaw).

After capture and field identification, all smolts were transferred to a flow-through 1.8 m diameter holding tank, supplied with 8 L/min of 10°C well water, and held for 1–2 days before tagging. Either a V9 or a V7 (VEMCO Ltd., Halifax, Nova Scotia, Canada) acoustic transmitter (frequency 69 kHz, 30–90 s ping rate) was implanted in each individual (see Table 1). Only smolts greater than 165 mm (for V9 tags) or 155 mm (for V7 tags) were selected for tagging to maintain a tag-to-body weight ratio of less than 7% for V9 tags and less than 5% for V7 tags. Each smolt was anesthetized in a bath of 70 mg/L MS-*222*, buffered to neutral pH, then placed on a v-shaped surgical stand equipped with a tube that administered a milder solution of anesthetic (MS-222 at 40 mg/L) over the gills. All surgical instruments and transmitters were soaked in ethyl alcohol and rinsed thoroughly with deionized water before use. Smolts were measured and weighed, and a small pelvic fin tissue sample was taken for DNA analysis. Incisions were made immediately anterior to the pelvic girdle, and the transmitter was placed within the body cavity parallel to the incision. The incision was then closed with two stitches using sterile monofilament sutures. Tagged smolts typically recovered within 2–3 min after being returned to freshwater, and were held in recovery tanks at the tagging site for 20–24 h before being released directly below the weir (rkm 0.05). No tagged smolt perished as a result of the surgeries and all appeared to be alert, behaving normally, and in good condition upon release.

**Table 1:** Tag type and length summary by year.

		Cutthroat	Hybrid	Steelhead
**2006**	**V7**	0	0	0
	**V9**	23	16	37
	**Length**	184±2	192±5	194±3
**2007**	**V7**	1	6	24
	**V9**	14	11	2
	**Length**	184±3	198±6	178±3
**2008**	**V7**	3	5	26
	**V9**	11	4	0
	**Length**	195±4	196±6	179±2
	**Total Number Tagged**	52	42	89

Number of each smolt type tagged with either V7 or V9 Vemco acoustic transmitters and each group's average length (± SE) for each year of the study. Only smolts greater than 155 mm were selected for tagging.

### Genetic Identification of Species and Hybrids

Smolts identified as steelhead were originally genotyped for 15 microsatellite DNA loci, using polymerase chain reactions (PCR) and capillary gel electrophoresis, as part of a study monitoring Hood Canal steelhead populations. During these initial analyses, we suspected that some of the putative steelhead samples were steelhead x cutthroat hybrids because for three of these loci (*Oke4* [37], *Ots3* [38], *Ots100* [39]), cutthroat trout showed genetic markers (alleles) that were distinguishable from those of steelhead. Putative steelhead smolts, for which we observed any cutthroat trout markers at these three loci, were further genotyped for four additional loci (*OCC-34*, *OCC-35*, *OCC-42*, *OM-47*) that were specifically developed to differentiate steelhead, cutthroat, and their hybrids [40]. All putative cutthroat smolts were also genotyped for these four diagnostic loci (genetic data available by request). We then calculated the hybrid index (I_H_) of each individual as described by Campton and Utter [17], using allele frequencies from these seven loci. The hybrid index is a value between 0.0 and 1.0 that indicates the level of hybridization of an individual. For our samples, a value of 0.0 would represent a pure cutthroat and a value of 1.0 would represent a pure steelhead. A first generation (F1) hybrid would be expected to have a value close to 0.5.

**Figure 1:**
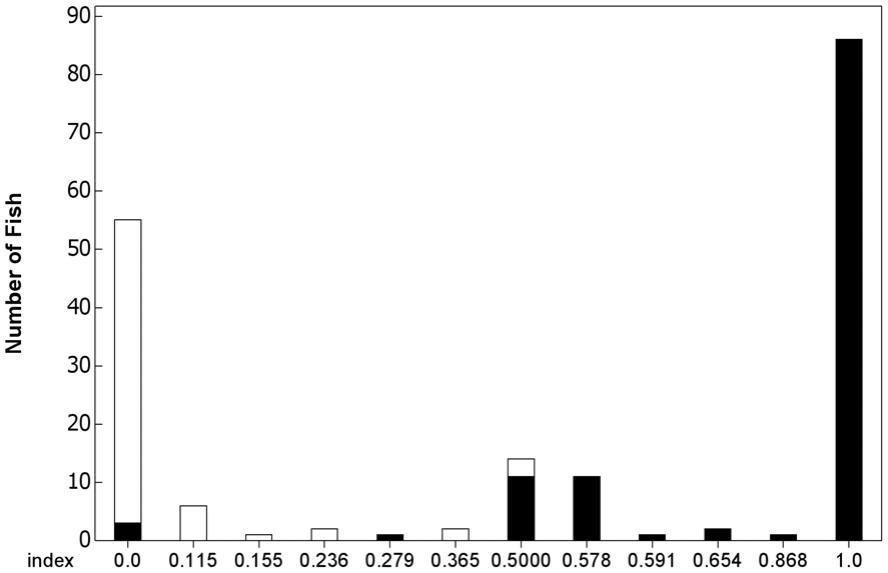
Hybrid Index Histogram. Numbers of phenotypic cutthroat (white bars) and phenotypic steelhead (black bars) classified along a continuum of hydrid indices ranging from 0.0 (pure cutthroat) to 1.0 (pure steelhead).

During the three study years, 14 of the 66 putative cutthroat smolts (21.2%) were genetically identified as hybrids, and 28 of the 117 putative steelhead smolts (23.9%) were identified as hybrids. Of the 14 phenotypic cutthroat hybrids, three had F1 hybridization patterns (0.4<I_H_ >0.6) - and 11 were likely second generation or greater (F1+). All of the phenotypic cutthroat F1+'s were likely cutthroat backcrosses (CT F1+; I_H_<0.4). A larger proportion of phenotypic steelhead hybrids were identified as F1 (23 out of 28). Only 4 F1+ hybrids were identified from the phenotypic steelhead hybrid group, and one individual was not able to be genotyped at enough loci to calculate I_H_. Of these phenotypic steelhead F1+ individuals, three had steelhead backcross (SH F1+; I_H_≥0.6) genotypes while the other one had a CT F1+ genotype. Three phenotypic steelhead were genetically identified as pure cutthroat (Figure 1).

### Smolt size

Steelhead smolts ranged in length from 159 to 236 mm. Hybrid and cutthroat smolts had similar size ranges (165–237 mm and 167–218 mm, respectively. Analysis of variance (ANOVA) was carried out to investigate differences in length by species (main effect) and by year (random effect). The interaction term (species x year) was also included. There were size differences between species in some years but not others (i.e., significant interaction term) (ANOVA; F_4,177_ = 6.01, P<0.001; Table 1). Mean sizes were not statistically different in 2006. Hybrids were significantly longer than steelhead and cutthroat in 2007, and both hybrids and cutthroat were longer than steelhead in 2008 (Tukey's multiple comparisons).

### Receiver Placement

**Figure 2:**
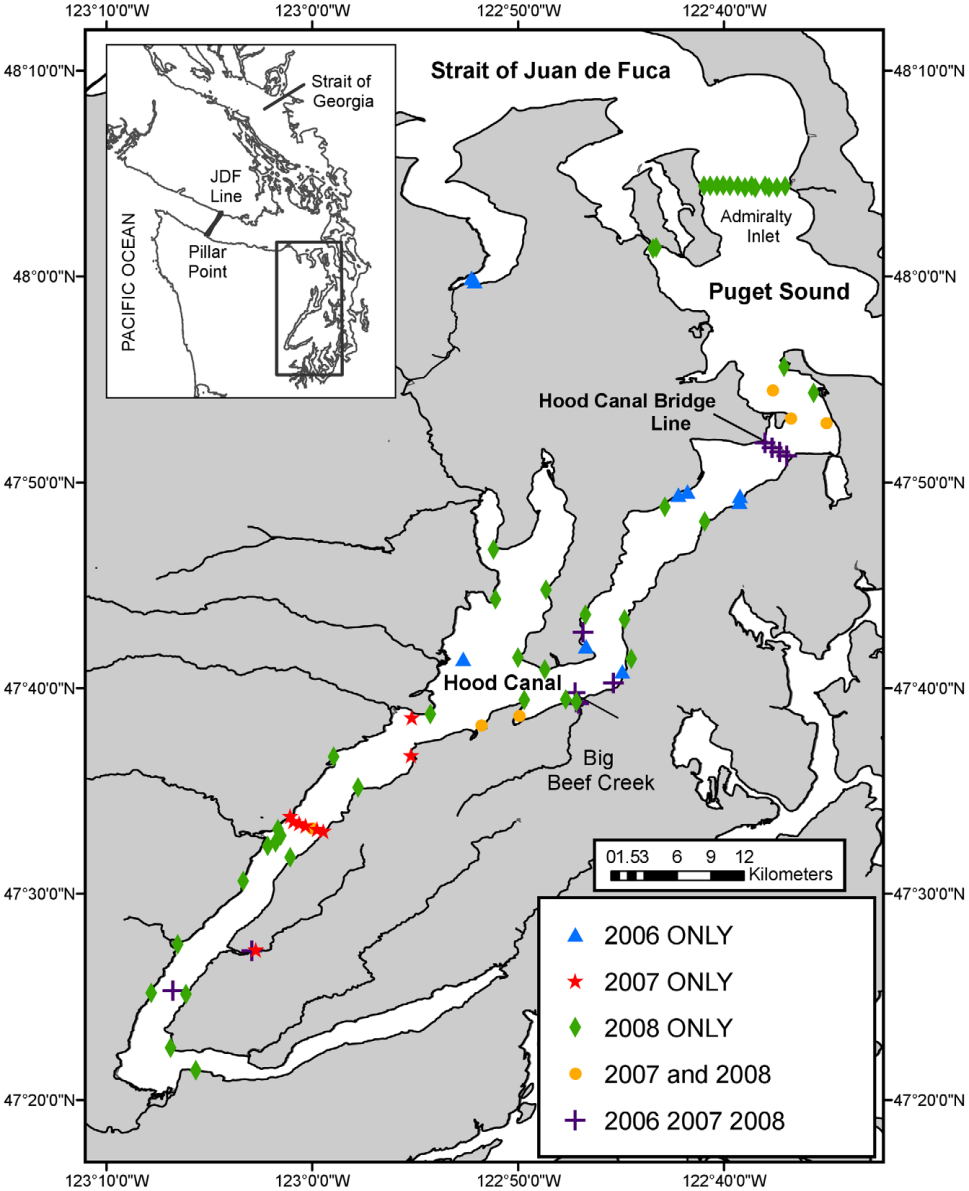
Locations of acoustic telemetry receivers in 2006, 2007, and 2008. In all three years, two receivers were placed at the river mouth to detect outmigrating smolts. The Hood Canal Bridge line was comprised of four receivers in 2006 and seven receivers in 2007 and 2008. A line of 31–33 receivers spanned the Strait of Juan de Fuca at Pillar Point in all three years, and a line of 13 receivers was deployed in Admiralty Inlet in 2008. Additional receivers were placed throughout Hood Canal and Puget Sound to observe movement patterns.

In 2006, 2007, and 2008, Vemco VR2 and VR2W acoustic receivers were deployed throughout Hood Canal in configurations varying by year, though many receiver locations were consistent (Figure 2). Receivers were placed with the intention of: i) detecting all smolts as they left Big Beef Creek and entered Hood Canal, (ii) detecting and recording the timing of smolts leaving Hood Canal, and (iii) capturing spatial and temporal movement patterns in the nearshore habitat within Hood Canal [34]. In all years, two receivers were placed in the Big Beef Creek estuary to obtain outmigration date and time. Four receivers were suspended from the Hood Canal floating bridge in 2006, spaced 580 m apart to accommodate the 400–500 m transmission radius of the V9 transmitters [41] (VEMCO Ltd., S. Tezak, unpublished data). In 2007 and 2008, seven receivers were suspended an average of 330 m apart along the bridge to ensure detection of the smaller and less powerful V7 transmitters, (range ∼200–300 m, VEMCO, Ltd.) [42]. Several single receivers were placed in shallow, nearshore locations and some mid-channel locations to achieve a broad spatial distribution of receivers in Hood Canal (Figure 2). To complement the fixed station receivers, boat surveys were occasionally performed (5 days in summer in 2006 (July–August), 12 days in 2007 (May–August), and 4 days in 2008 (July–August)) to determine fish position up to 10-km north and south of the Big Beef Creek estuary. The Pacific Ocean Shelf Tracking Project (POST) [43] provided detection data from their compatible acoustic arrays, including 30–31 receivers (spaced 750–800 m apart) located at Pillar Point in the Strait of Juan de Fuca (2006–2008) and a 13-receiver line (spaced 250–500 m apart) across Admiralty Inlet within Puget Sound (2008) (Figure 2).

### Data Analysis

Detection data were used to reconstruct the migration track made by each fish as it moved between receivers. Both spatial (total travel distance, tortuosity (defined below), dispersal distance) and temporal (residence time, estuarine residence, dispersal time) components of each track were quantified for each species. Total travel distance, tortuosity, residence time, and estuarine residence time were each calculated from only those receivers in Hood Canal. Dispersal time and distance were calculated using data from the Hood Canal and POST receivers. Track analysis software, written by Jose Reyes-Tomassini (publicly available, contact: Jose.ReyesTomassini@noaa.gov), was used to calculate a tortuosity index and the total travel distance. Tortuosity describes the extent to which a track meanders, defined here as:
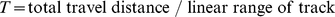
where the total travel distance was the sum of all track segments, and the linear range of the track was the distance between the two most distant receivers included in the track (i.e., often the distance between the northernmost and southernmost receiver in the track). Estuarine residence time was the time between the first and last detections at either of the two Big Beef Creek estuary receivers. Hood Canal residence time (hereafter ‘residence time’) was the time between the last detection at a Big Beef Creek estuary receiver and the last detection at any receiver within Hood Canal. Residence times were also reported in Moore et al. [34] for Big Beef Creek steelhead smolts, however the residence times presented here were recalculated using different receiver detections (final Hood Canal detection as opposed to final Hood Canal Bridge detection). The analysis presented here also includes 2008 outmigrants that were not included in Moore et al. [34]. Dispersal time was the time between the first detection at a Big Beef Creek estuary receiver and a smolt's last detection at any receiver (i.e., either within Hood Canal, at the Admiralty Inlet array, or at the Strait of Juan de Fuca array), and dispersal distance was defined as the distance from the head of the Big Beef Creek estuary (capture and release site) to the location of the last detection at any receiver, with negative distances representing southern movement within Hood Canal, and positive distances representing northern and seaward movement.

Multivariate analyses of track parameters were performed to compare migration patterns between species, starting with total travel distance (TD), tortuosity (T), residence time (RT), and estuary time (ET) as candidate variables. Fish either not detected or detected for <24 h on an estuary receiver were excluded from analysis. Regression analysis of hybrid behavior indicated that only one (tortuosity) out of the four behavioral parameters was dependent on I_H_, (TD: T = −0.017, p = 0.865; T: T = −2.42, p = 0.21; RT: T = 1.27, p = 0.213: ET: T = 0.47, p = 0.641), so data for all hybrids were pooled into one category for analysis. All variables were square-root or log_10_ transformed to improve normality and homogeneity of variance. The transformed variables were then screened for multicollinearity. Total travel distance and tortuosity were highly correlated (r = 0.606), as were travel distance and residence time (r = 0.479) but the remaining combinations of variables were less so (r<0. 344). In a principle component analysis (TD + T + RT + ET ∼ species), total travel distance explained less of the variation than did tortuosity in the first principle component, and was therefore dropped from multivariate analysis. Differences in the migratory behaviors of cutthroat, steelhead, and hybrid smolts were investigated using multivariate analysis of variance (MANOVA), with species as the main effect and tortuosity, residence time, and estuary time as response variables. Multiple comparisons were then carried out to determine pairwise differences between cutthroat, steelhead and hybrid behavior.

Since the transmission life and detection range of the V7 and V9 tag types differed (by ∼30 days and transmission range by ∼200 m (VEMCO, Ltd.)), some of the response variables could be biased, therefore MANOVA was carried out to test for these effects. Detections of smolts with V9 tags recorded after 96 days, which was the longest residence time recorded with a V7 tag, were excluded from the analysis. Then, effects of tag type and species (main effects) on residence time, tortuosity, and estuary time in 2007 and 2008 (years during which tag type differed) were investigated. No effect of tag type was found (F_tag type_ = 0.26, df = 1, 109, P = 0.613). Receiver configurations also differed between years, so MANOVA was carried out again to test species and year (main effects) against the same response variables: residence time, tortuosity, and estuary time. No significant year effect was found (F_year_ = 2.54, df = 2, 150, P = 0.083), and similar proportions of the different species groups were tagged within each year (see Table 1), so data from all years and all tag types were pooled.

## Results

**Figure 3:**
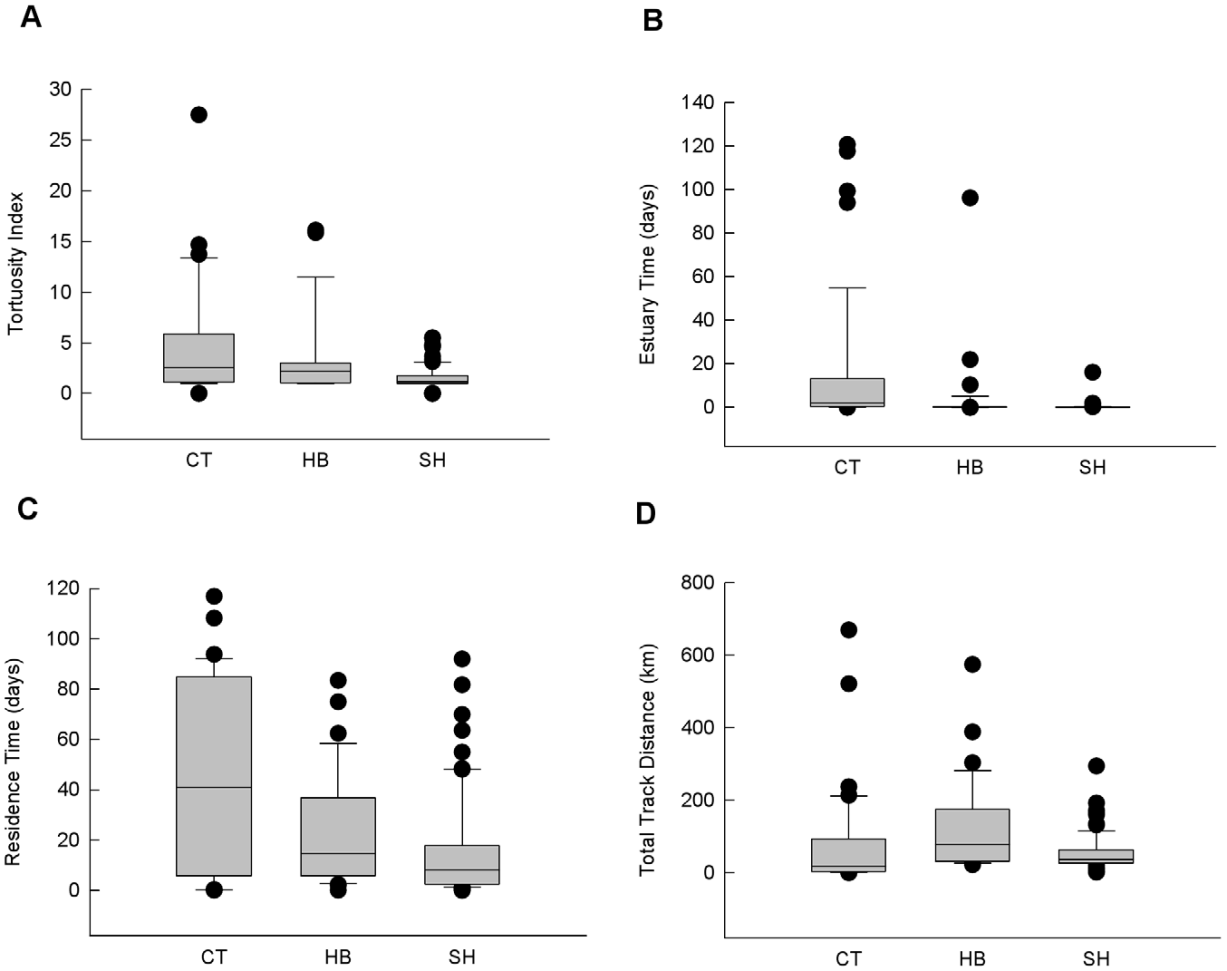
Box and whisker plots (median, interquartile range, data range, outliers) of migratory track parameters. (A) residence time (days), the time between ocean entry and last detection, (B) estuary time (days), the time between first and last estuary detection, (C) total track distance (kilometers), the sum of all track segments, and (D) the tortuosity index, which measures the extent to which a track meanders, and is the sum of all track segments divided by the linear distance between the two farthest receivers included in the track.

Overall, species had a significant effect on migratory behavior, as defined by estuarine residence time, tortuosity, and residence time parameters (MANOVA; F_2,148_ = 0.414, P<0.001). Multiple comparison analysis revealed significant mean differences in behavior between steelhead and cutthroat smolts (F_1,112_ = 0.497, P≤0.001), between steelhead and hybrids (F_1,108_ = 0.149, P = 0.001), and between cutthroat and hybrid smolts (F_1,74_ = 0.303, P = 0.001). Cutthroat smolts exhibited the longest median residence time in the Hood Canal (41 days), followed by 15 days for hybrids and only 8 days for steelhead (Figure 3A). Median estuarine residence times also differed (cutthroat  = 2.7 days, hybrids  = 6.7 hours, steelhead  = 1.1 hours; Figure 3B). Median total track distance measured only 19.5 km for cutthroat, compared to 60.4 km for hybrids, and 34.0 km for steelhead (Figure 3C). Cutthroat and hybrid smolt tracks had similar median tortuosity indexes (2.5 and 2.2, respectively), whereas steelhead smolts had straighter, more direct tracks (median T  = 1.2; Figure 3D). Three out of the four track parameters measured for hybrid smolts were intermediate between cutthroat and steelhead values (residence time, tortuosity, and estuarine residence time; Figure 3), but hybrids had longer track distances than either cutthroat or steelhead smolts. Data from all four parameters were variable and contained several outliers, indicating the overall diversity of behaviors within the three species. However, steelhead had the narrowest range of measurements for all four track parameters (Figure 3A–D).

**Figure 4:**
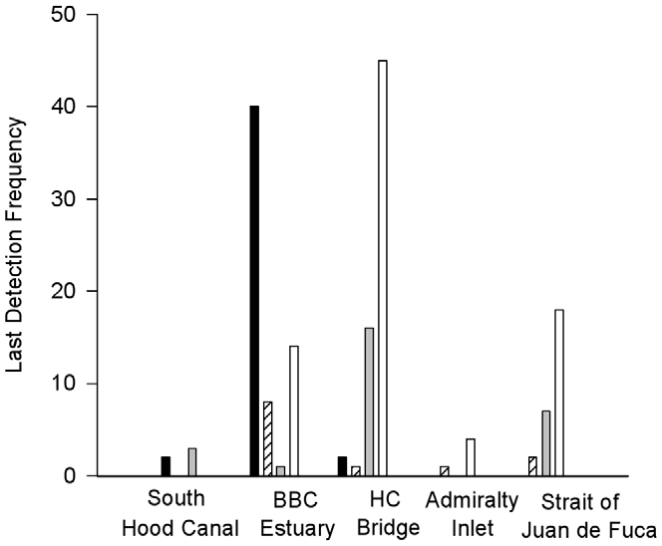
Numbers of cutthroat, steelhead, and hybrid smolts last detected at locations within the study area. South Hood Canal  = >20 km south of the Big Beef Creek estuary, Estuary  =  within 20 km of the Big Beef Creek estuary, HC Bridge  =  within 10 kilometers of the Hood Canal Bridge, Admiralty inlet  = 50 km from estuary, Strait of Juan de Fuca  = 150 km from estuary). Cutthroat (−Ι_H_ = 0.0) are represented by black bars, phenotypic cutthroat hybrids (−Ι _H_ = 0.270) by cross-hatched bars, phenotypic steelhead hybrids (−Ι _H_ = 0.543) by gray bars, and steelhead (−Ι _H_ = 1.0) by white bars.

**Figure 5:**
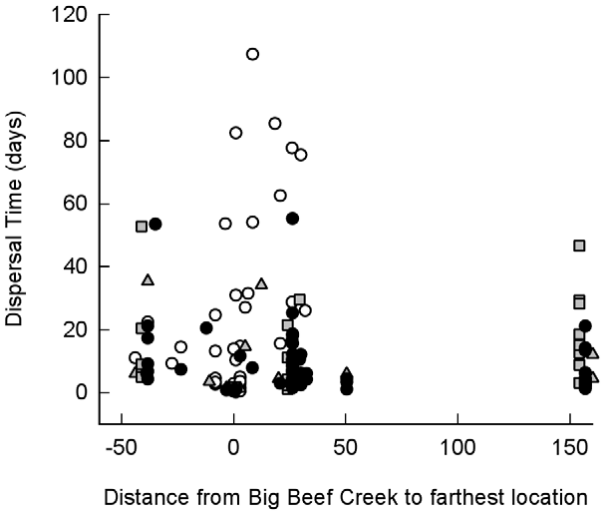
Dispersal time plotted against distance from Big Beef Creek to each smolt's farthest detection location. Dispersal time is the time between the last estuary detection and the farthest detection. Negative distances represent movement to southern locations, and positive distances represent movement to northern locations. Locations of cutthroat smolts are represented by dark circles, phenotypic cutthroat hybrids are represented by shaded triangles, phenotypic steelhead hybrids are represented by shaded squares, and steelhead are represented by white circles. Locations of some fish were changed slightly to accommodate viewing of all symbols.

Hybrids exhibited distribution patterns that overlapped with the cutthroat and steelhead patterns (Figures 4 and 5). No obvious differences in distribution were apparent between phenotypic steelhead hybrids and phenotypic cutthroat hybrids. At the end of each track, most steelhead smolts (80%) were located near or north of the Hood Canal exit, while most cutthroat (89%) grouped within 20 kilometers of the estuary (Figure 4). Many hybrid smolts (70%) were last detected, along with two large groups of steelhead smolts, at either the Hood Canal Bridge receiver line (+27 km from Big Beef Creek) or the Strait of Juan de Fuca receiver line (+157 km from Big Beef Creek). This behavior indicated fish migrating to the ocean, as would be expected for steelhead. Of the individuals that were last located at the Strait of Juan de Fuca, hybrids took a significantly longer time to travel from the estuary to those receivers than did steelhead (Welch's t-test: t_11_ = 3.53, P = 0.005; hybrid median  = 21.5 days, steelhead median  = 10.5 days). Three hybrid smolts (7.5%) and two cutthroat smolts (4.5%), but no steelhead smolts, were last located nearly 40 km south of the Big Beef Creek estuary, after varying periods of time (Figure 4).

Hybrid smolts displayed dispersal patterns that overlapped with those of both cutthroat and steelhead (Figure 5). The hybrids represented a large proportion of the smolts whose farthest detection was the Strait of Juan de Fuca line (56%), and the hybrids also represented a large proportion (47%) of the group who travelled nearly 50 km south of their estuary of origin. Cutthroat smolts exhibited a wide range of dispersal patterns within Hood Canal, while the majority of steelhead smolts predictably dispersed at least as far as the Hood Canal Bridge (Figure 5).

## Discussion

Marine migration patterns of steelhead and cutthroat have been well-documented and are quite different [25], [31], [44]. Steelhead leave the nearshore and coastal waters rapidly and feed on the high seas for typically 2 or 3 years, whereas cutthroat tend to remain in estuaries and nearshore coastal waters, especially in close proximity to shore, and generally do not over-winter at sea but return annually to freshwater. This study provides evidence of additive genetic control over migration behavior, as steelhead x cutthroat hybrids exhibited behavior intermediate to that of either species. Three important migration parameters, (1) residence time, (2) estuary time, and (3) tortuosity index, were all intermediate for hybrid smolts in relation to pure smolts. MANOVA analysis indicated a distinct grouping of hybrid individuals based on these behavioral components of migration, suggesting that hybrids, as a group, behaved differently than either of the parental species. The differences in body size were slight and so could not readily explain the differences in behavior.

Migratory behavior of steelhead and cutthroat has evolved to optimize growth and survival in relation to different environmental and ecological constraints, thus sudden disruption of evolved patterns due to hybridization may be maladaptive. Hawkins [18] documented both intermediate swimming ability and intermediate aggressive tendencies in cutthroat x steelhead hybrids, and noted that these differences may put hybrids at a disadvantage when competing for stream habitat with steelhead. However, the freshwater ecology of the species is more similar than the marine ecology, so the reduction of hybrid fitness in freshwater may be less than that in marine waters, where intermediate migration behaviors may reduce hybrid fitness relative to pure species. With influence from cutthroat genes, ocean-bound hybrids may spend more time in the estuary and thus reach the ocean at an inopportune time, missing the optimal ‘window’ for ocean entry [45]. Influence from steelhead genes may cause near-shore dwelling hybrids to leave favorable rearing areas such as Hood Canal but not reach the open ocean feeding grounds occupied by steelhead.

On the other hand, variation in migration patterns, facilitated through genetic exchange, may provide the means for hybrids to exploit resources unavailable to parental populations, especially if annual environmental conditions vary considerably. Grant and Grant [4] provided evidence for this hypothesis in a study of Darwin's finches, finding that hybrids of two pure finch species had higher fitness in certain environmental conditions, while pure species prospered in others.

Hybrids had longer track distances than did cutthroat or steelhead, which may affect mortality rates in the Hood Canal. Long hybrid track distances paired with intermediate tortuosity indexes resulted in long, directed movements within the Hood Canal. A large proportion of hybrids (33%) was detected nearly 50 kilometers south of the Big Beef Creek estuary, while a much smaller proportion of steelhead (9.6%) went that far south. Many of these hybrid smolts then migrated the entire length of Hood Canal and were later detected at the Hood Canal Bridge (46%) or the Strait of Juan de Fuca (38%). Steelhead smolt marine mortality appears to depend on distance travelled [34], so long migrations undertaken by hybrid smolts may impose higher mortality rates than those suffered by pure species.

At the end of each track, hybrids were distributed either near the exits to Hood Canal or Puget Sound (Hood Canal Bridge or Strait of Juan de Fuca), as would be expected for steelhead, or close to or south of the Big Beef Creek estuary, more characteristic of cutthroat. This result suggested that they eventually displayed one of the pure species' options (i.e., either long migration to the Pacific Ocean or short nearshore movements near the natal stream). There was no dominant pattern; similar proportions of hybrids displayed each pattern. Moreover, phenotypic cutthroat hybrids did not necessarily always exhibit migration patterns similar to pure cutthroat, nor did phenotypic steelhead hybrids behave like steelhead. The apparent mismatch between behavior and morphology may present another hybrid disadvantage. A physiological example of this type of mismatch in cutthroat x steelhead hybrids was documented by Hawkins and Foote [46], who observed incongruence between paternally conferred rates of egg development and maternally conferred yolk size in hybrid offspring.

The typical limitations of acoustic telemetry affected the results of this study to some extent. Values of all of the behavioral parameters measured depended on the number of receivers and their locations. However, receivers were placed with previous knowledge of characteristic migration paths for both species, and the study was done “blind” with respect to the hybrids because they were only identified after the fact. There were a few fish that were not detected after only a few days, and it is unclear if these fish migrated to another area or died, so some of the smolt tracks measured in this study were shorter than others. We did not find any clear mortalities of fish within 10-km of BBC (from mobile tracking), but the great majority of smolts die at sea [44], and it is likely that this was the fate of many of these fish with short duration tracks. The possibility that a fish was in the vicinity of a receiver and not detected because of transmitter malfunction can not be ruled out but this would not explain the differences in patterns between the pure species and hybrids.

Much of the uncertainty surrounding the nature of steelhead x cutthroat hybridization stems from the unknown fitness of hybrids relative to pure species in the wild. The relative fitness of the F1 progeny determines whether occasional hybridization strengthens isolating mechanisms or leads to genetic introgression [47]. Bettles et al. [28] measured survival of hybrid juveniles in relation to pure steelhead and cutthroat in 13 British Columbia streams, and found lower proportions of age-1 hybrids than age-0 hybrids in two streams but no clear patterns in the other streams. Young et al. [29] assessed hybridization rates of Big Beef Creek steelhead smolts in 1996, and found that 4 out of 18 steelhead (22.2%) had genotypes characteristic of hybrids. Ten years later, we found a similar rate of hybridization (23.9%) in the Big Beef Creek steelhead population, indicating that further introgression had not occurred. This may indicate that hybrids are less fit than parental species, and have been unable to survive to spawn in large numbers. Further evidence of limited hybrid survival is the low numbers of F1+ individuals present in the steelhead and cutthroat sampled in this study. In the absence of selection, the ratio of F1 hybrids to first generation backcrosses to second generation backcrosses should be 1∶2∶4 [48]. Therefore the ratio of F1s to all backcrosses would have to be 1∶6 to assume no fitness differences, and the ratio found in this study was 1∶1.3, suggesting lower hybrid survival at some life history stage.

Many more cutthroat backcrosses (CT F1+) than steelhead backcrosses (SH F1+) were found in Big Beef Creek during the study. It is possible that some of the F1+ hybrids are actually F2 hybrids (hybrid x hybrid), but even taking this possibility into account, the pattern of hybrids backcrossing to cutthroat and rarely to steelhead is notable. In Abernathy Creek in southwest Washington, Kennedy et al. [49] similarly observed more F1 smolts than backcrosses and no steelhead backcrosses. Young et al. [29] found fewer backcrosses than F1s in a survey of creeks in Washington, though other studies have documented a majority of backcrosses (California: [23], Washington and Oregon: [20]). These inconsistent results may indicate variability in the fitness of F1 and backcrossed hybrids, and/or they could reflect differences in the life history stage or stream location at which samples were taken.

Campton and Utter [17] suggested that the marine phase, and specifically migratory disorientation, was a possible limiting factor in the production of hybrids, hence the low incidence of hybrid adults observed in the wild. The novel migration patterns of hybrids observed in this study show a behavioral divergence from pure species' patterns of migration. This divergence may be the mechanism by which hybrids experience higher rates of mortality. Little direct evidence is available to confirm this hypothesis, though data from one year of adult steelhead microsatellite analysis (n = 29, same analysis methods as this study) on Big Beef Creek showed no adult phenotypic steelhead hybrids in 2007 (Donald Van Doornik, unpublished data). The fact that anadromous populations of steelhead and cutthroat exhibit much lower levels of introgression than resident rainbow trout and inland cutthroat trout also supports the marine phase limitation theory. Most streams along the US west coast known to contain cutthroat x steelhead hybrids report hybridization levels less than 36% [22]–[24], [49] (though see [20] and [28] for exceptions), while hybrid swarms are the norm in inland populations of sympatric rainbow and cutthroat [10], [50], [51]. Further investigation of hybrid smolt-to-adult survival is needed to determine whether or not the marine phase limits hybrid introgression.

Whether hybridization between coastal cutthroat trout and steelhead is natural or facilitated by human activities is important to consider, and the answer may determine how populations are managed. Rhymer and Simberloff [1] cite habitat modification, fragmentation, and introduced species as major contributors to hybridization, and maintain that the magnitude of the hybridization problem has generally been underestimated. A significant correlation was found between level of habitat degradation and rates of hybridization in 30 sympatric trout populations on Vancouver Island [52], but on the other hand, extensive hybridization between steelhead and coastal cutthroat has been documented in a pristine area of Alaska [24]. Whether human modification of US west coast streams has led to steelhead x cutthroat hybridization is debatable and difficult to test, but in light of recent environmental change and continued population growth it is more important than ever to answer such questions.

Hood Canal steelhead are part of the Puget Sound Evolutionarily Significant Unit listed as threatened under the Endangered Species Act, and Big Beef Creek steelhead and cutthroat runs are assumed to be a fraction of their former size. Hybridization between these species may represent a response to low population size. At low population densities, mating skew tends to decrease as a result of less male-male mate competition than is present at high densities, and females become less choosy [53]. In effect, low densities then perhaps lead to relaxed sexual selection and increased probability of interspecific matings. Previously reproductively isolated cichlid species interbred when sexual selection was relaxed as a result of difficulty in finding mates due to turbid water conditions [54]. If steelhead and cutthroat are having difficulty finding or synchronizing spawn timing with conspecifics, a better alternative to failing to spawn may be to spawn with a member of a closely related species. Reduced conspecific male-male competition may also lead to increased success of interspecific sneak-spawning tactics.

In summary, cutthroat x steelhead hybrid smolts exhibit migration behaviors intermediate to pure parental species. When specific parameters are grouped, these intermediate behaviors form novel migration strategies. The divergence of hybrid migration behavior may be maladaptive compared to the strategies of parental populations which have evolved locally over time, yet it is possible that new migration strategies may be beneficial in a variable or changing environment. Big Beef Creek cutthroat x steelhead hybridization rates seem to be somewhat stable at around 20–25%, with no evidence of a hybrid swarm, indicating the probability of a limiting factor at some point in the life history of hybrids. The data presented here support the theory that the marine phase limits hybrid production; testing this would involve genetic analysis of returning adults to determine if the proportion of hybrids decreased from that seen among the smolts. More comparative studies are needed to determine the fitness of hybrids relative to pure species, and to identify the anthropogenic or natural causes of cutthroat x steelhead hybridization on the US West Coast.
