# Correction: Early Marine Migration Patterns of Wild Coastal Cutthroat Trout (*Oncorhynchus clarki clarki*), Steelhead Trout (*Oncorhynchus mykiss*), and Their Hybrids

**DOI:** 10.1371/annotation/89faa149-4569-4b03-a073-9ac3aeed86cd

**Published:** 2010-10-06

**Authors:** Megan E. Moore, Fred A. Goetz, Donald M. Van Doornik, Eugene P. Tezak, Thomas P. Quinn, Jose J. Reyes-Tomassini, Barry A. Berejikian

The caption for figure 5 contains an error in the key; groups of fish were assigned the wrong symbols. The correct caption is: Figure 5. Dispersal time plotted against distance from Big Beef Creek to each smolt's farthest detection location. Dispersal time is the time between the last estuary detection and the farthest detection. Negative distances represent movement to southern locations, and positive distances represent movement to northern locations. Locations of cutthroat smolts are represented by white circles, phenotypic cutthroat hybrids are represented by shaded triangles, phenotypic steelhead hybrids are represented by shaded squares, and steelhead are represented by dark circles. Locations of some fish were changed slightly to accommodate viewing of all symbols. 

